# Exploring soil microbial and plant parasitic nematode communities involved in the apple replant disease complex in Nova Scotia

**DOI:** 10.1038/s41598-025-17349-8

**Published:** 2025-10-02

**Authors:** Shawkat Ali, Keith D. Fuller, Svetlana N. Yurgel, Tom Forge, Vicky Lévesque, Mark Mazzola

**Affiliations:** 1 Agriculture and Agri-Food Canada, Kentville Research and Development Centre, Kentville, NS B4N 1J5 Canada; 2 United States Department of Agriculture (USDA), Agricultural Research Service, Grain Legume Genetics and Physiology Research Unit, Prosser, WA 99350 USA; 3 Agriculture and Agri-Food Canada, Summerland Research and Development Centre, Summerland, BC V0H 1Z0 Canada; 4 Department of Plant Pathology, Stellenbosch University, Private Bag X1, Matieland, 7600 South Africa

**Keywords:** Apple replant disease (ARD), Soil microbiome, Next-generation sequencing, Soil pathogens, Fungi, Oomycete, *Malus domestica*, Biotechnology, Microbiology, Plant sciences

## Abstract

Apple replant disease (ARD) is incited by a complex of causal agents including various fungi, oomycetes, and plant parasitic nematodes. These causal agents can differ significantly in abundance between orchard sites within a geographic region. Knowledge of the specific etiology of ARD is required in order to develop commercially viable soil management strategies to combat specific/individual components of the pathogen complex. In this study, we analyzed soil from six ARD affected orchard sites to assess the presence and composition of fungal, bacterial and oomycetes communities, as well as the prevalence of plant parasitic nematodes. Five fungal, and 17 bacterial classes were differentially represented in the soil microbiomes across the different locations. Mortierellomycetes was the most abundant fungal taxa represented followed by Sordariomycetes. *Mortierella exigua*, a fungal endophyte, was the most abundant fungal amplicon sequence variant (ASV) in the core microbiome. Among bacteria, Proteobacteria was the most prevalent phylum identified in these orchard soils. Several potential phytopathogenic fungi associated with ARD, as well as endophytes including *Fusarium oxysporum*,* F. solani*, *Nectria ramulariae*,* Ilyonectria robusta* and *Nectriaceae*, were identified in ARD soils. Among oomycetes, *Pythium attrantheridium* (*Globisporangium attrantheridium*), and *P. irregulare* (*Globisporangium irregulare*) were the most abundant taxa. Additionally, six different groups of plant-parasitic nematodes were found across the ARD orchard soils. Root-lesion nematodes, *Pratylenchus* spp., which are commonly associated with ARD, were identified in all orchard soils at population densities range from 12 to 33/100 cm^3^ soil. This research enhances our understanding of the ARD pathogen complex and provide important insights for developing alternative disease management strategies in the apple industry.

**Supplementary Information:**

The online version contains supplementary material available at 10.1038/s41598-025-17349-8.

## Introduction

Apple replant disease (ARD) occurs as a result of the activity of pathogenic microorganisms and nematodes that have accumulated in orchard soils and subsequently infect newly planted trees into these soils during orchard renovation. ARD is common in nearly all apple and other pome fruit growing regions of the world. Young apple trees affected by ARD have symptoms such as uneven growth, stunting, root necrosis, shortened internodes above ground, deformed leaves, reduced root biomass, and reduced yield compared with trees free of the disease^1^. In the past, pre-plant application of broad spectrum soil fumigants was employed effectively to control ARD around the world^2^. Traditional soil fumigants, such as methyl bromide, 1,3-dichloropropene and chloropicrin have a biologically broad spectrum of activity and thus eradicate pathogenic and non-pathogenic microorganisms indiscriminately. Safety and environmental concerns over methyl bromide led to international legislation and a ban on methyl bromide in 2005. The 1,3-dichloropropene and chloropicrin had been one of the main pre-plant soil fumigants used in Canada following the phase-out of methyl bromide. With the recent ban on the use of 1,3-dichloropropene and chloropicrin in Canada, ARD has re-emerged as a major threat to the successful re-establishment of apple orchards in Canada. Thus, there is an urgent need to develop alternative, more specific and sustainable approaches with reduced chemical inputs for control of this biologically complex disease. Potential alternative disease control methods include biofumigation, anaerobic soil disinfestation (ASD), incorporation of organic amendments, semi-selective agrochemicals, and immunization with antagonistic and beneficial microorganisms^3–5^. Understanding the of the specific etiology of ARD at a particular site is required to develop alternative, commercially viable soil management strategies to combat specific/individual components of the disease complex.

Various biotic factors such as an increased pathogen populations, a decline in beneficial microbes, dysbiosis of the soil microbial community and disrupted microbial network that reduces soil disease suppressiveness along with abiotic factors such as nutrient imbalance in the soil, toxic root exudates or microbial metabolites, poor drainage and soil compaction have all been associated with the decline in apple yield and quality in ARD-affected sites^6–8^. However most recent research suggests ARD is primarily caused by biotic factors as soil disinfection through pasteurization and fumigation with broad-spectrum chemical have significantly reduced the disease compared to untreated soils^9–13^. A number of studies have demonstrated that ARD is incited by an interacting complex of various fungal and oomycete pathogens, and plant parasitic nematodes^14–16^. The relative abundance of these entities and differences at the species level within any specific group often differ between sites within a geographic region^17,18^. Braun^19,20^ identified *Pythium irregulare* (*Globisporangium irregulare*) and *Cylindrocarpon lucidum* in combination as causal pathogens of ARD in five old apple orchard soils of the Annapolis Valley, Nova Scotia, Canada. In Europe, *Cylindrocarpon* like fungi (*Ilyonectria* spp. and *Thelonectria* sp.) were reported as major fungal pathogens contributing to ARD in Germany, Austria and Italy, while *Pythium* spp. were found as major ARD causal pathogens only in Germany^21^. In addition, several studies based on inoculation and isolation of fungi and oomycetes from symptomatic plants established the hypothesis that ARD is a complex of fungal (*Cylindrocarpon/Nectria*,* Rhizoctonia*) and oomycete (*Pythium*,* Phytophthora*) species^3,22^. In another study in South Africa, Van Schoor et al.^17^ isolated *Cylindrocarpon*,* Pythium* and *Fusarium* spp. from lesions in apple roots grown in six orchard soils, but the pathogenicity of these isolates was not confirmed in plant inoculations. These authors also assigned secondary roles to *Rhizoctonia* and plant parasitic nematodes *Pratylenchus* and *Xiphinema* spp. as causal agents of ARD. In Washington State, oomycetes such as *Pythium* spp. and *Phytophthora cactorum* along with fungal pathogens such as *Rhizoctonia solani* and *Cylindrocarpon destructanans* have consistently been isolated and identified as major causal agent of ARD^13^.

Several biotic factors—including fungal and oomycetes pathogens as well as lesion nematodes have been shown to be implicated in ARD^17^. However, the relative abundance, diversity, dominance and even the presence of these organisms can vary significantly across different geographical regions^10,13,23^. Reports on the involvement of these microbes causing ARD on their own, or in combination are inconsistent especially regarding the role of plant parasitic nematodes *Pratylenchus penetrans*^3,6,24^. The involvement of bacterial pathogens in ARD is debatable and is not well investigated when compared with fungi and oomycetes. However, bacteria belonging to *Actinomycetes*, *Bacillus* and *Pseudomonas* might also be a part of the disease complex^25^. Composition of the *Pseudomonas* spp. community was shown to vary significantly between soils that were suppressive or conducive to ARD^26^. Also, some species of *Streptomyces* have been reported to reduce the incidence of *Rhizoctonia* root rot infections in apple^27^.

Although fungal and oomycete pathogens have traditionally been associated with ARD, the conventional approach of isolating, identifying, and reinoculating these pathogens may have inadvertently downplayed the potential role of the broader soil microbial communities in development or suppression of ARD. It is now well established that the structure, diversity, and composition of soil microbial communities are critical to ecosystem functioning, soil health, and plant growth^28,29^. Advances in next-generation sequencing technologies have enabled comprehensive analysis of the entire microbiome present in soil samples, allowing us to better understand the full suite of microbial taxa involved in soil and plant health dynamics. However the composition and response of fungal, bacterial, oomycete and nematode communities involved in ARD have not yet been fully elucidated at ARD sites in Nova Scotia apple growing regions. Therefore the aims of the present study were to (i) compare the abundance and composition of the broader soil fungal, bacterial and oomycete communities across six ARD orchard sites in the Annapolis Valley of Nova Scotia; (ii) identify the relative abundance of potential fungal and/or oomycete pathogens associated with ARD in these soils, and (iii) further assess the contribution of plant parasitic nematodes at ARD sites in orchard soils. To achieve these objectives, we used Illumina Miseq deep sequencing approach by targeting ITS2 for fungi, V6-V8 region of 16 S rRNA for bacteria and ITS region for oomycetes on soil samples collected from six ARD sites. For nematode isolation and quantification in soil samples, we used wet sieving-sucrose centrifugation procedure followed by counting and identification to the genus level based on morphological characteristics.

## Results

### Orchard site characteristics

The physio-chemical properties of six orchard soils used in this study were characterized (Tables 1 and 2). In Nova Scotia apples are produced on soils with a relatively narrow range of soil textures that are generally sandy to coarse loamy (Table 2). In these soils, water retention and internal drainage (Table 2) can often be better correlated with silt plus clay content rather than clay alone. This is in contrast to some orchard soils that occur in other apple growing regions of the world. Based on plant bioassays conducted in the respective orchard soils (Table 2), five of the six soils were ranked as having the potential for severe ARD development with only one orchard soil (CAN), as likely experiencing a moderate level of disease severity (Table 2). The CAN site has been an orchard for the fewest number of years (Table 2), while the site with the highest potential for ARD severity (BER) has the longest history of apple production.

**Table 1:** Soil fertility parameters of six apple orchards in the Annapolis Valley of Nova scotia.

Orchard sites*	OM**	pH	*P*_2_0_5_	K_2_0	Ca	Mg	Al	CEC	Ca	Mg	K	H
	%	H_2_0	kg ha^− 1^					***	%	%	%	%
ROW	3.7	5.9	1097	275	2913	171	1571	10.2	71.7	7.0	2.8	18.7
BER	3.2	6.4	856	523	2863	455	1516	11.8	60.6	16.1	4.7	18.1
AYL	2.6	6.2	497	194	1715	382	1585	8.1	52.5	19.5	2.4	25.1
KEN	4.1	6.6	848	425	3885	512	1442	14.8	65.5	14.3	3.0	16.9
CAN	3.2	5.4	970	665	2239	520	1555	11.9	46.9	18.1	6.1	28.6
ROE	3.7	5.9	1097	275	2913	171	1571	10.2	71.1	7.0	2.8	18.7

*Orchard sites: ROW, Rockland West area; BER, Berwick area; AYL, Aylesford area; KEN, Kentville area; CAN, Canard area; ROE, Rockland East area. **OM,  organic matter; ***CEC, cation exchange capacity meq 100 g^− 1^.

### Fungal and bacterial microbiome composition

**Fig. 1:**
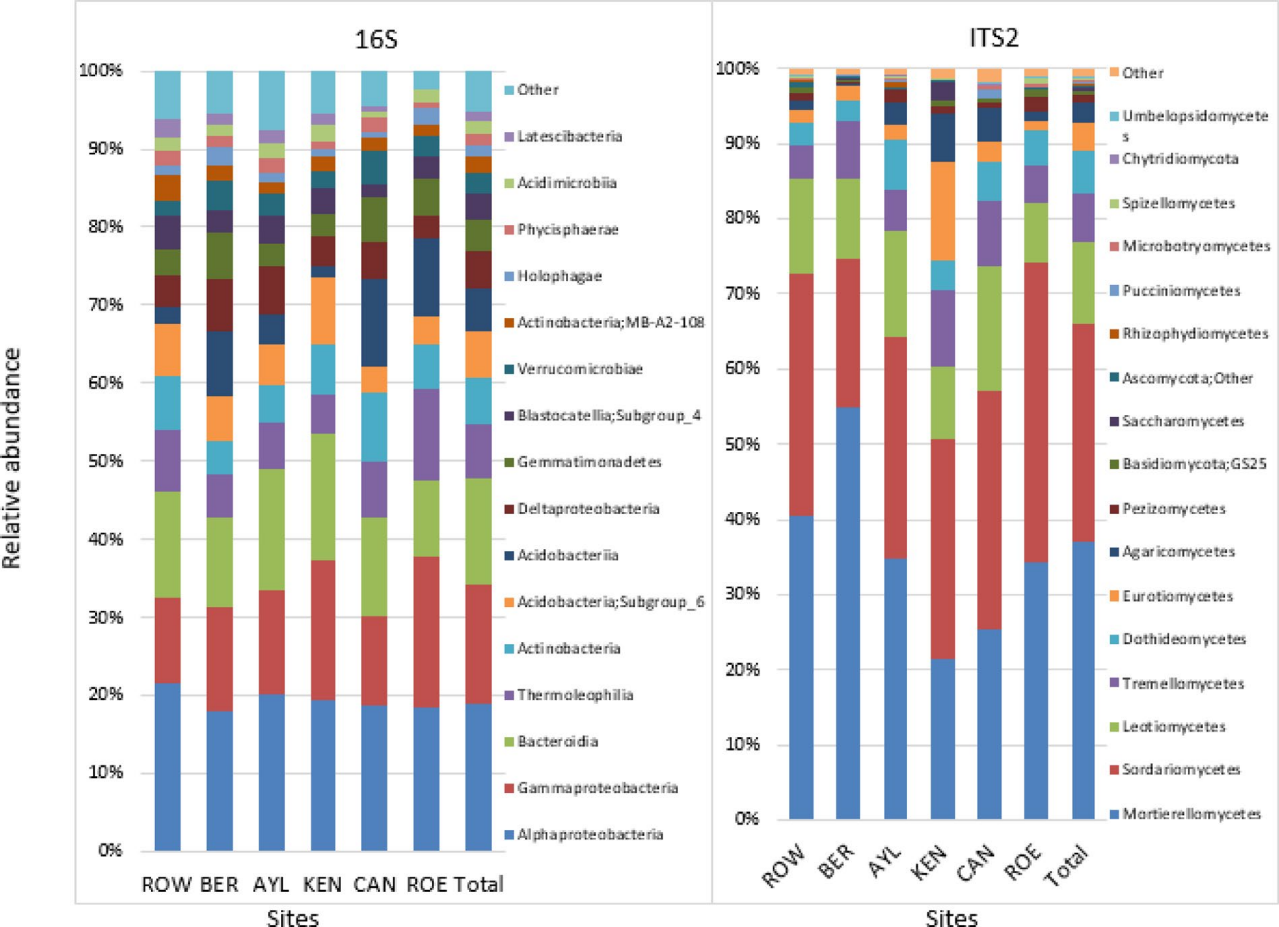
Relative abundance of soil bacterial and fungal community structure across six Nova Scotia apple orchards. Bacterial (left panel) and fungal (right panel) annotated at class level and represented by at least 1% of total 16 S RNA and 0.1% of total ITS2 reads are shown. Orchard locations in the Annapolis Valley of Nova Scotia in Canada: ROE, Rockland East area; ROW, Rockland West area; CAN, Canard area; AYL, Aylesford area; KEN, Kentville area; BER, Berwick area.

ITS2 and 16 S rRNA (V6-V8 region) amplicon sequencing was performed to evaluate orchard soil fungal and bacterial communities, respectively. Taxonomic analysis revealed that Ascomycota had the highest relative abundance among fungal phyla in the orchard soils comprising 48% of all ITS2 reads identified in the study, followed by Zygomycota, (37%) and Basidiomycota (10%) (Fig. 1 right panel). Mortierellomycetes, Sordariomycetes, Leotiomycetes, Tremellomycetes, Dothideomycetes, Eurotiomycetes, and Agaricomycetes were the most abundant fungal classes found in the ITS2 derived soil microbiome and were represented by 37%, 29%, 11%, 6%, 6%, 4%, and 3% of total ITS2 rRNA reads, respectively (Fig. 1 right panel). Proteobacteria was the most prevalent phylum identified in orchard soils (38% of total 16 S rRNA reads) with two classes Alphaproteobacteria (19%) and Gammaproteobacteria (15%) having the highest relative abundance among bacterial taxa (Fig. 1 left panel). Bacteroidetes (Bacteroidia) was the third most relatively abundant class represented by 14% of total 16 S rRNA reads. Actinobacteria and Acidobacteria were also highly represented at 16% of total 16 S rRNA reads, each. Actinobacteria classes exhibiting the highest relative abundance included Thermoleophilia (8% of total 16 S rRNA reads) and Actinobacteria (6%), while Subgroup6 (6% ) and Acidobacteriia (5%) were present at the greatest relative abundance among Acidobacteria classes (Fig. 1 left panel).

### Variation of fungal and bacterial microbiome composition across apple replant orchard soils

The analysis of strength and statistical significance of sample groupings (Permutational Multivariate Analysis of Variance Using Distance Matrices (PERMANOVA) based on weighted unifrac beta-diversity distances with 999 permutations) indicated a significant variation in community composition across different ARD orchard soils (p value < 0.001). Approximately 55% of fungal variation and 54% of bacterial community variation were explained by the sampling site (orchard location) (data not shown). However, we did not detect a significant difference in fungal or bacterial community alpha-diversity among sites (p value > 0.05; data not shown). Redundancy Analysis (RDA) indicated that that soil paraments such as P_2_0_5_, K_2_0, organic matter (OM) and pH, as well as disease severity had significant effects on both bacterial and fungal microbiome, although these effects were relatively minor. For the bacterial community OM and disease severity were the most influential factors, explaining 6% and 5% of community variation, respectively (Fig. S1). Similarly, approximately 6% of fungal community variation was explained by these factors.

Three fungal and three bacterial classes differed significantly in relative abundance between the six ARD orchard soils (Fig. S2). Eurotiomycetes and Saccharomycetes were overrepresented in KEN, while Spizellomycetes were overrepresented in ROE, compared with other locations (Fig. S2). Acidobacteriia were over-represented in BER and CAN orchards, while unidentified Latescibacteria were detected at the lowest relative abundance in ROE soil compared with other locations, and Bacilli were over represented in CAN compared with all other sites (Fig. S2).

**Fig. 2:**
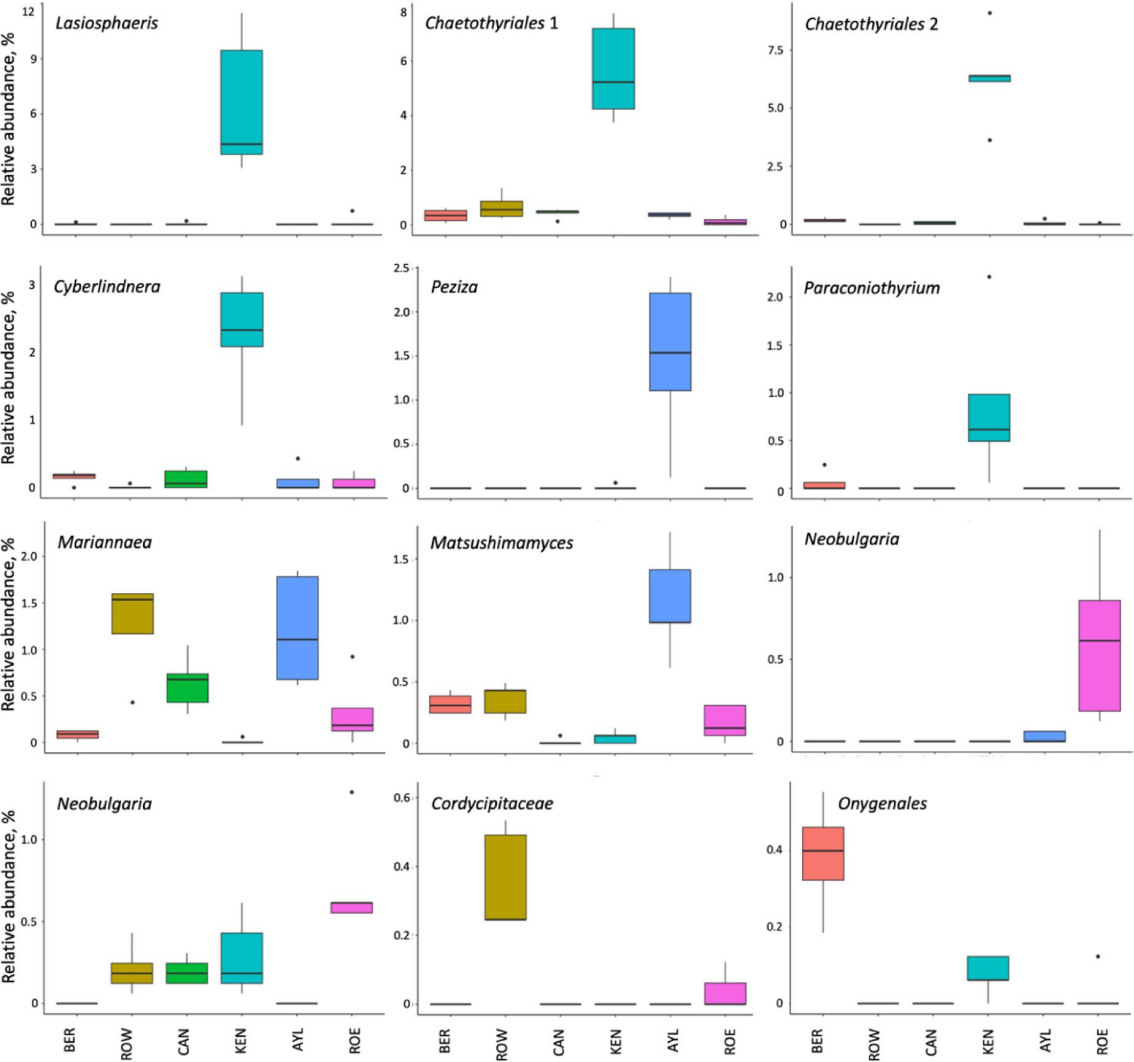
Boxplots showing the differentially relative abundance of fungal taxa at class level across six Nova apple Scotia orchards. Based on ANCOM test with 1% FDR. Orchard locations in the Annapolis Valley of Nova Scotia, Canada: ROE; Rockland East area; ROW, Rockland West area; CAN, Canard area; AYL, Aylesford area; KEN, Kentville area; BER, Berwick area.

**Fig. 3:**
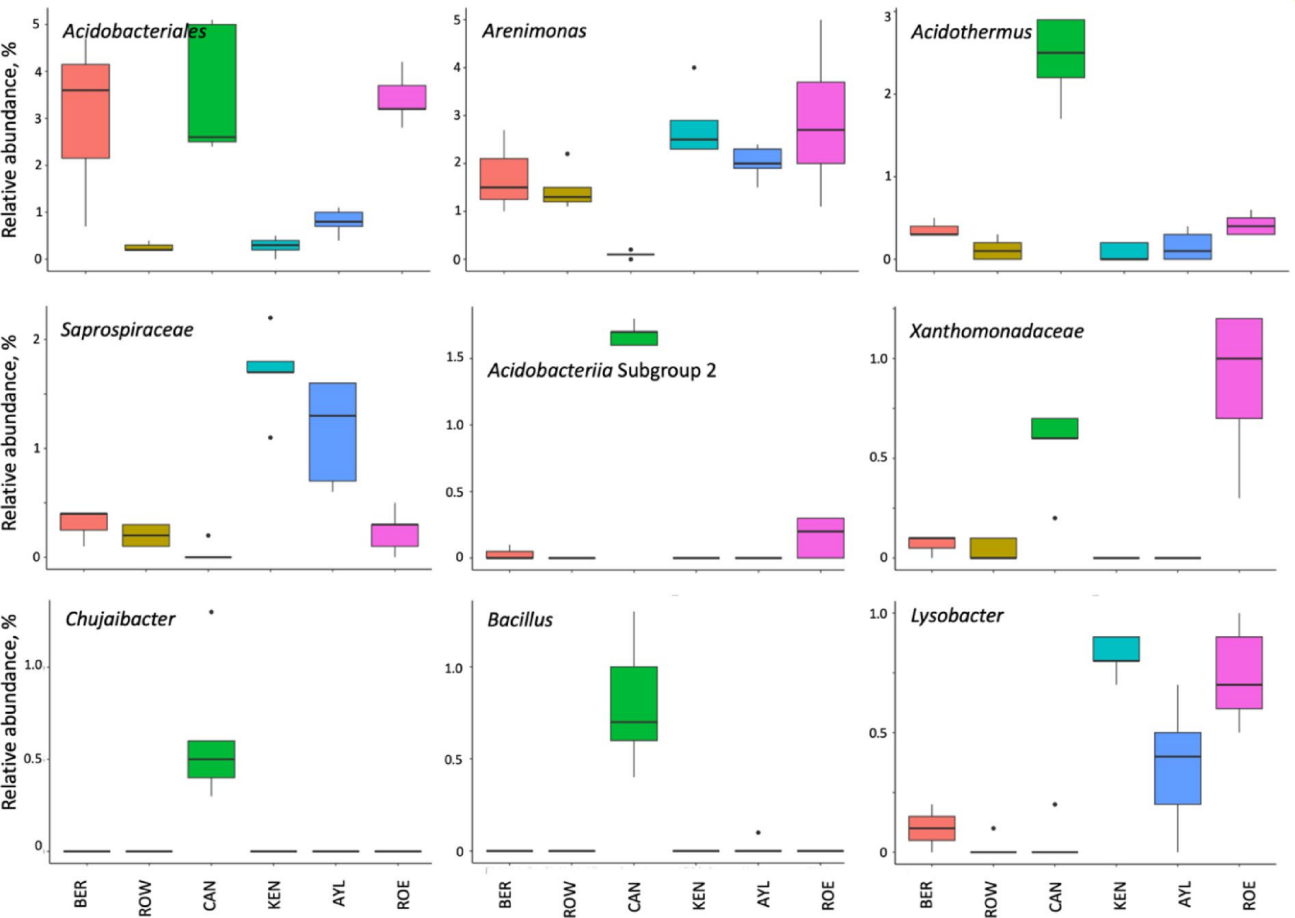
Boxplots showing the differentially relative abundance of soil bacterial taxa at class level across six Nova Scotia apple orchards. Based on ANCOM test with 1% FDR. Orchard locations in the Annapolis Valley of Nova Scotia, Canada: ROE, Rockland East area; ROW, Rockland West area; CAN, Canard area; AYL, Aylesford area; KEN, Kentville area; BER, Berwick area.

At the genus level, we identified 12 fungal and eight bacterial taxa differentially represented between orchards. Fungal taxa *Lasiosphaeris*, two unidentified Eurotiomycetes (*Chaetothyriales* 1, *Chaetothyriales* 2 ), *Cyberlindnera*, and *Paraconiothyrium* were overrepresented in KEN soil (Fig. 2), while *Peziza*,* Mariannaera* and *Matsushimamyces* were more abundant in AYL (Fig. 2). For bacterial taxa, *Acidothermus*,* Acidobacteria* Subgroup 2, *Chujaibacter* and *Bacillus* were most abundant in CAN soil (Fig. 3).

### Core fungal and bacterial ASVs

We extended our analysis to identify core bacterial and fungal ASVs that were consistently found across communities from all six apple orchards and detected in at least 80% of all soil samples analyzed in this study. We identified 29 fungal and 15 bacterial ASVs, represented by 7% and 50% of total ITS2 and 16 S rRNA reads, respectively (Tables S1, S2). *Mortierella exigua* was the most abundant fungal ASV in the core microbiome, 9% of total ITS2 reads. A number of potential phytopathogenic/endophytic ASVs were a part of the core microbiome including *Fusarium oxysporum* (4%), *Fusarium solani* (1%), *Nectria ramulariae* (synonym *Cylindrocarpon ehrenbergii*) (0.5%) and *Ilyonectria robusta* (*Cylindrocarpon*-like asexual morphs) (1%), as well as *Nectriaceae* (0.4%). Bacterial ASVs within this group were affiliated with Acidobacteria (4 ASVs), Bacteroidetes (4 ASVs) and Proteobacteria (5 ASVs). The *Xanthobacteraceae* family and *Pseudolabrys* were the most abundant ASVs represented by 14% of total 16 S rRNA reads.

A total of 44 bacterial and 15 fungal families were found in at least 80% of the samples from each orchard (Tables 3 and 4). These families were represented by 74% and 73% of total ITS2 and 16 S rRNA reads, respectively. Core fungal families detected at high relative abundance included Mortierellaceae, Nectriaceae, Helotiaceae, Chaetomiaceae and Piskurozymaceae, represented by 36%, 12%, 5%, 5%, and 4% of total ITS2 reads, respectively (Table 4). Similarly, core bacterial families Chitinophagaceae, Xanthobacteraceae, Nitrosomonadaceae, Xanthomonadaceae, and Sphingomonadaceae had high relative abundances in the total bacterial microbiome and were represented by 10%, 8%, 5%, 4%, and 3% of total 16 S rRNA reads, respectively (Table 3).

**Table 2:** Soil physical properties and characteristics of six experimental apple orchards in the Annapolis Valley of Nova Scotia.

Orchard sites*	Soil texture	Drainage class**	Soil EAM*** (mm/30 cm)	Year planted	Rootstock/variety****	Density trees ha^− 1^	Row width (m)	ARD severity (% R)*****
ROW	Sandy	4	34.6	1989	M26/RD	683	6.1	235.0
BER	Coarse-Loamy	3	23.2	1986	MM111/RC	236	6.5	267.4
AYL	Coarse-Loamy	2	37.4	1964	M111/Mac	494	5.7	117.9
KEN	Coarse-Loamy	4	18.5	1987	M26/Mac	694	4.8	166.8
CAN	Coarse Loamy	5	27.3	2008	M9/GD	2574	4.3	66.3
ROE	Sandy	4	12.6	1972	M111/Mac	249	7.3	168.5

* Orchard sites: ROW (Rockland West area), BER, Berwick area; AYL, Aylesford area; KEN, Kentville area; CAN, Canard area; ROE, Rockland East area; ** Drainage class: 5 = rapidly drained, 4 = well drained, 3 = moderately well drained, 2 = imperfectly drained; *** EAM = easily available moisture; ****RD: Red Delicious; RC: Royal Cort; Mac: MacIntosh; GD: Golden Delicious; M26, M111, M9: apple rootstocks of the Malling series; *****ARD Severity (%) = percentage response of apple replant disease, severity measured in a standard greenhouse bioassay.

**Table 3:** Core bacterial ASVs (family level) found in at least 80% in all soil samples collected in six Apple orchards located in the Annapolis Valley of Nova scotia.

Family	Mean rel. freq.
Bacteroidetes; Bacteroidia; Chitinophagales; Chitinophagaceae	10%
Proteobacteria; Alphaproteobacteria; Rhizobiales; Xanthobacteraceae	8%
Acidobacteria; Subgroup_6; Other	5%
Proteobacteria; Gammaproteobacteria; Betaproteobacteriales; Nitrosomonadaceae	5%
Gemmatimonadetes; Gemmatimonadetes; Gemmatimonadales; Gemmatimonadaceae	4%
Proteobacteria; Gammaproteobacteria; Xanthomonadales; Xanthomonadaceae	4%
Proteobacteria; Alphaproteobacteria; Sphingomonadales; Sphingomonadaceae	3%
Actinobacteria; Thermoleophilia; Gaiellales; uncultured	3%
Acidobacteria; Acidobacteriia; Acidobacteriales; uncultured	2%
Proteobacteria; Gammaproteobacteria; Xanthomonadales; Rhodanobacteraceae	2%
Actinobacteria; Thermoleophilia; Gaiellales; Gaiellaceae	2%
Acidobacteria; Blastocatellia (Subgroup 4); Pyrinomonadales; Pyrinomonadaceae	2%
Verrucomicrobia; Verrucomicrobiae; Chthoniobacterales; Chthoniobacteraceae	2%
Acidobacteria; Acidobacteriia; Solibacterales; Solibacteraceae (Subgroup 3)	1%
Planctomycetes; _Phycisphaerae; _Tepidisphaerales; _WD2101_soil_group	1%
Actinobacteria; MB-A2-108; Other; Other	1%
Proteobacteria; Alphaproteobacteria; Caulobacterales; Caulobacteraceae	1%
Proteobacteria; Gammaproteobacteria; Gammaproteobacteria Incertae Sedis	1%
Actinobacteria; Actinobacteria; Propionibacteriales; Nocardioidaceae	1%
Actinobacteria; Thermoleophilia; Solirubrobacterales; 67 − 14	1%
Acidobacteria; Blastocatellia (Subgroup 4); Blastocatellales; Blastocatellaceae	1%
Bacteroidetes; Bacteroidia; Cytophagales; Microscillaceae	1%
Actinobacteria; Actinobacteria; Corynebacteriales; Mycobacteriaceae	1%
Actinobacteria; Actinobacteria; Pseudonocardiales; Pseudonocardiaceae	1%
Proteobacteria; Deltaproteobacteria; Myxococcales; Haliangiaceae	1%
Proteobacteria; Alphaproteobacteria; Rhizobiales; Hyphomicrobiaceae	1%
Proteobacteria; Deltaproteobacteria; Desulfarculales; Desulfarculaceae	1%
Proteobacteria; Alphaproteobacteria; Rhizobiales; Rhizobiales Incertae Sedis	1%
Verrucomicrobia; Verrucomicrobiae; Pedosphaerales; Pedosphaeraceae	1%
Actinobacteria; Actinobacteria; Micromonosporales; Micromonosporaceae	1%
Acidobacteria; Holophagae; Subgroup 7;Other	1%
Other; Other; Other; Other	1%
Proteobacteria; Alphaproteobacteria; Rhizobiales; Rhizobiaceae	1%
Actinobacteria; Thermoleophilia; Gaiellales; Other	1%
Proteobacteria; Gammaproteobacteria; Betaproteobacteriales; TRA3-20	1%
Actinobacteria; Thermoleophilia; Solirubrobacterales; Solirubrobacteraceae	1%
Actinobacteria; Acidimicrobiia; IMCC26256; Other	1%
Proteobacteria; Alphaproteobacteria; uncultured; Other	< 1%
Proteobacteria; Alphaproteobacteria; Rhizobiales; Beijerinckiaceae	< 1%
Bacteroidetes; _Bacteroidia; _Chitinophagales; _uncultured	< 1%
Nitrospirae; Nitrospira; Nitrospirales; Nitrospiraceae	< 1%
Actinobacteria; _Actinobacteria; Frankiales; Nakamurellaceae	< 1%
Rokubacteria; NC10; Rokubacteriales; Other	< 1%
Proteobacteria; Alphaproteobacteria; Elsterales; URHD0088	< 1%

*Mean rel. freq., mean relative frequency.

**Table 4:** Core fungal ASVs (family level) found in at least 80% of all soil samples collected in six Apple orchards located in the Annapolis Valley of Nova scotia.

Family	Mean rel. freq.
Mortierellomycota; Mortierellomycetes; Mortierellales; Mortierellaceae	36%
Ascomycota; Sordariomycetes; Hypocreales; Nectriaceae	12%
Ascomycota; Leotiomycetes; Helotiales; Helotiaceae	5%
Ascomycota; Sordariomycetes; Sordariales; Chaetomiaceae	5%
Ascomycota; Leotiomycetes; Helotiales; Helotiales fam Incertae sedis	4%
Basidiomycota; Tremellomycetes; Filobasidiales; Piskurozymaceae	4%
Ascomycota; Sordariomycetes; Hypocreales; Hypocreaceae	2%
Ascomycota; Sordariomycetes; Glomerellales; Plectosphaerellaceae	2%
Basidiomycota; Tremellomycetes; Cystofilobasidiales; Mrakiaceae	1%
Ascomycota; Sordariomycetes; Hypocreales; Clavicipitaceae	1%
Basidiomycota; Tremellomycetes; Tremellales; Trimorphomycetaceae	1%
Ascomycota; Eurotiomycetes; Chaetothyriales; Herpotrichiellaceae	1%
Ascomycota; Sordariomycetes; Hypocreales; Bionectriaceae	1%
Unassigned; Other; Other; Other; Other	< 1%
Ascomycota; Sordariomycetes; Hypocreales; Hypocreales fam Incertae sedis	< 1%

*Mean rel. freq., mean relative frequency.

### Composition of oomycetal microbiome

**Fig. 4:**
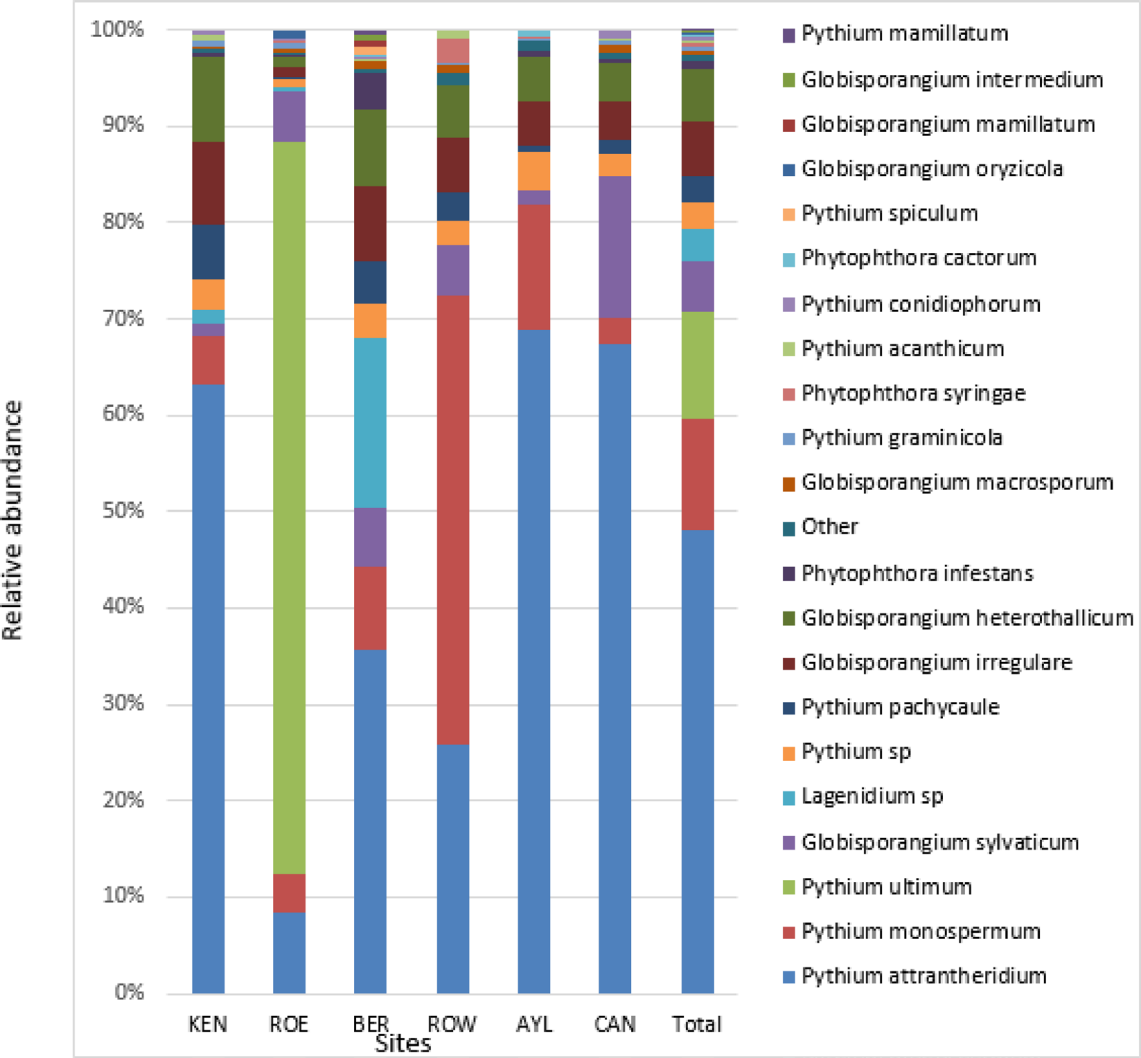
Relative abundance of soil oomycetes community structure across six Nova Scotia apple orchards. Oomycete annotated at lass level and represented by at least 1% of total ITS reads are shown. Orchard locations in the Annapolis Valley of Nova Scotia, Canada: ROE, Rockland East area; ROW, Rockland West area; CAN, Canard area; AYL, Aylesford area; KEN, Kentville area; BER, Berwick area. *P. attrantheridium* (*Globisporangium attrantheridium*), *P. monospermum* (*Nematosporangium monospermum*),* P. ultimum* (*Globisporangium ultimum*) and *P. irregulare* (*Globisporangium irregulare*).

**Fig. 5:**
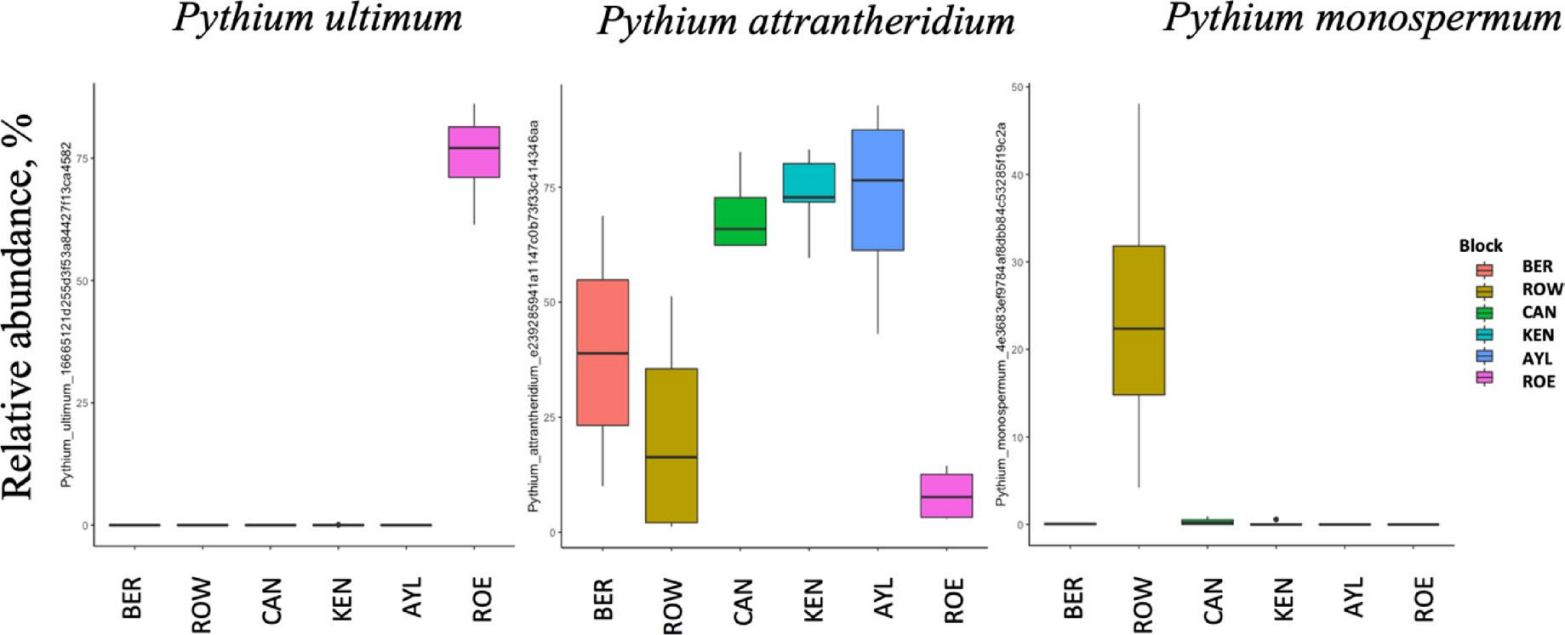
Boxplots showing the differentially relative abundance of soil oomycetes taxa at specie level across six Nova Scotia apple orchards.Based on ANCOM test with 1% FDR. Orchard locations in the Annapolis Valley of Nova Scotia, Canada: ROE, Rockland East area; ROW, Rockland West area; CAN, Canard area; AYL, Aylesford area; KEN, Kentville area; BER, Berwick area. *P. ultimum* (*Globisporangium ultimum*),* P. attrantheridium* (*Globisporangium attrantheridium*) and *P. monospermum* (*Nematosporangium monospermum*).

*Pythium attrantheridium* (*Globisporangium attrantheridium*), *P. monospermum* (*Nematosporangium monospermum*), and *P. ultimum* (*Globisporangium ultimum*) were the most abundant oomycetal taxa identified in the analysis. They were represented by 50%, 12% and 12% of total oomITS reads, respectively (Fig. 4). While the communities did not differ in Shannon diversity between the orchards (p value > 0.05; data not shown), location was a strong factor affecting oomycetal community structure. More than 60% (p value < 0.001) of the community variation was explained by sampling site (data not shown). This variation in community structure was reflected in the visual differences in the community profiles across the orchards (Fig. 4). *P. attrantheridium*,* P. monospermum* and *P. ultimum* exhibited the most pronounced apparent variation across location. *P. attrantheridium* is present across all six sites while *P. monospermum* and *P. ultimum* are over represented at only one site. This variation was attributed to significant difference in relative abundances of three ASVs, which uniquely represented these species (Fig. 5). ASV, annotated as *P. ultimum*, was predominantly found in ROE soils and was represented by around 75% of total ROE oomITS reads. ASVs, annotated as *P. attrantheridium*, was overrepresented in CAN, KEN and AYL soil and *P. monospermum* ASV was predominantly found in ROW soil, comprising more than 20% of total ROW oomITS reads (Fig. 5).

### Nematode analysis

Among the nematode communities extracted from orchard soils, six potential groups of plant parasitic nematodes were identified. from the orchard soils: Root-lesion (*Pratylenchus* spp.), dagger (*Xiphinema* spp.), ring (criconematidae), pin (paratylenchidae), spiral (hoplolaimidae) and root-knot (*Meloidogyne* spp.) (Table 5). Root-lesion nematodes were found at all six sites, at population densities ranging from 12 to 33 *Pratylenchus*/100 cm^3^ soil (Table 5). Dagger nematodes were found in five of the six sites and were recovered at relatively high population densities, e.g.>100 *Xiphinema*/100 cm^3^ soil, in two of the sites (Table 5). All dagger nematodes observed had morphological characteristics conforming to the *X. americanum*-complex of species.

**Table 5:** Population densities (nematodes/ 100 cm^3^ soil) of genera of plant-parasitic nematodes extracted from six Apple orchards in the Annapolis Valley of Nova scotia.

Orchard sites*	Nematodes / 100 cm^3^ soil					
	*Pratylenchus*	*Xiphinema*	*Mesocriconema*	*Paratylenchus*	*Hoplolaimidae*	*Meloidogyne*
ROW	14	149	21	0	2	0
BER	13	172	2	0	13	0
CAN	14	40	1	0	2	0
KEN	33	3	10	30	9	15
AYL	18	3	21	10	7	4
ROE	12	0	9	2	2	0

*Orchard sites: *ROW*, Rockland West area; BER, Berwick area; AYL, Aylesford area; KEN, Kentville area; CAN, Canard area; ROE, Rockland East area.

## Discussion

The etiology of ARD is challenging to elucidate as, unlike many other soilborne diseases, numerous causal agents have been isolated from affected sites in different parts of the world. ARD has been reported by many as a complex of different types of microbial pathogens and plant parasitic nematodes which can differ in their relative abundances between sites within a geographic region^3,6,13,22,30^. Some of the previous replant disease studies consider soil fertility and nutrient availability as major contributors to the disease^31^. But other studies have negated this hypothesis as fertilization, and an increase in soil nutrients was not able to eliminate ARD^18,32^. In this study, we measured several soil fertility parameters, such as OM, available P, K, and several other essential nutrients for plant growth at the six ARD sites. None of them were significantly correlated with the severity of ARD. However, there were no “Low” disease severity sites identified in this study, only sites with “Moderate” or “Severe” disease potential was identified. These results are in agreement with a study carried out in Bohai Gulf China by Gongshuai et al.^32^ as they also did not find any direct correlation between ARD severity and soil nutrient composition. Ad hoc field observations in the Nova Scotia apple industry suggest that summer drought stress can aggravate the severity of the disease. Similar observations have been made in New York State (Rosenberger, 2023 personal communication). However, no correlation was observed between probable ARD severity and soil chemical properties, as well as the physical soil parameters linked with potential moisture stress, such as soil texture, drainage class, and plant available moisture (Table 2). Although the soil properties didn’t influence the severity of the ARD, the longevity of the apple production likely affected it as demonstrated by the RDA analysis.This study provides evidence that ARD in Annapolis Valley is of biological origin as all apple growth parameters measured in pasteurized soil from all sites were markedly improved when compared with non-pasteurized soil in a greenhouse bioassay, a finding that is consistent with previous studies reporting ARD as being of a biological nature^6,32,33^.

Soil microbial communities and soil biodiversity play a crucial role in soil ecosystem functions and plant health by decomposition of dead organic materials, nutrient recycling, nutrient availability to plants, suppression of soilborne diseases and inducing plant disease resistance^29,34,35^. Having a soil with a wide variety of microorganisms help to create a more competitive environment that can inhibit the growth and invasion of harmful pathogens^36^. We detected considerable variation in soil bacterial and fungal community composition and structure across ARD orchard sites. More than 50% variation was observed in bacterial and fungal community composition across the orchard study sites. Also, although three bacterial and three fungal classes were differentially represented among the orchard soils, we did not observe any correlation between severity of ARD and fungal or bacterial alpha-diversity across the six orchard sites. This again could have resulted from the lack of a “Low” ARD potential orchard soil included in this study.

The soils of these six orchards exhibited a consistent core microbiome. The results showed that, Ascomycota, Zygomycota and Basidiomycota were the fungal phyla that were most identified at the ARD sites that accounted for 95% of the total ITS2 reads. Ascomycota was revealed as the most dominant phylum present in ARD sites tested in this study. This finding is similar to previous studies that also reported Ascomycota as the most dominant phylum in ARD sites^24,32^. The class Mortierellomycetes exhibited the greatest relative abundance in the ITS2 microbiome. Species of *Mortierella* have been reported as saprophytes and some of them belong to the plant growh-promoting fungi. I another study abundance of *Mortierella* spp. has been reported in healthy banana soil^38^. In apple orchards, some studies have indicated that *Mortierella* spp. have a mutualistic relationship with apple tree and have been associated negatively with the severity of ARD^32^ and are positively related with plant growth^24^. In another study by Soman et al. 1999 ^39^reported three metabolites from *Mortetierella vinacea* with antifungal and antibacterial activity suggesting an important role for this fungus in suppressing soil-borne pathogens associated with ARD.

*Fusarium oxysporum* and *Fusarium solani* were the most relatively abundant core ASV in this study. *Fusarium* is a large genus that contains saprophytes, endophytes, and plant and animal pathogens. In soil, *Fusarium* is often associated with plant debris and most species are saprophytic and relatively abundant in the soil microbial community. Kelderer et al.^22^ reported that *F. solani* and *F. oxysporum* were the most frequently isolated species followed by *Cylindrocarpon* spp. binucleate *Rhizoctonia* spp. and *Fusarium* spp. from apple replant orchards soil in Italy. No pathogenicity assays were performed for *Fusarium* spp. in their study as *Fusarium* spp. were considered to be non-pathogenic on apple^22^. Several species of the genus *Fusarium* also produce bioactive secondary metabolites that mediate positive interaction with host plants^40,41^. Both *F. solani* and *F. oxysporum* are considered endophytes as they are usually isolated from asymptomatic root tissue with high abundance, not only from apple tree but several other crops^3,13,18,23,42,43^. In an orchard field study, *Fusarium* spp. were recovered at a greater frequency from the roots of apple trees grown in methyl bromide fumigated soils than the roots of apple trees cultivated in non-treated ARD soil at the same site^13^. However, more than 20 species of the genus *Fusarium* are pathogens of higher plants causing root rot, vascular wilt, and storage rot^44^. *Fusarium* is often isolated from both diseased and healthy apple tree roots, but most isolates representing several species did not prove to be pathogenic on apples^13,45^. Tewoldemedhin et al. (2011)^3^ frequently isolated *Fusarium* from all orchards in the study, but most proved to be non-pathogenic towards apple seedlings and only two of the isolates (*F. avenaceum* and *F. solani*) were only weakly virulent on apple seedlings. Other studies have shown that *F. solani* was either non-pathogenic, or had low virulence towards apple seedlings^13,18^. We also detected several other fungal genera in this study, such as *Ilyonectria*,* Nectria* and *Nectriaceae*, which fall into the group that is traditionally called *Cylindrocarpon*-like fungi^46^. These genera are reportedly negatively involved in apple growth^20,24,47^. Our findings are in agreement with these previous studies in identifying *Cylindrocarpon* as a causal agent of ARD Braun^20^.

*Pythium* spp. have long been reported on a global basis as contributing pathogen to ARD. However, numerous of studies have demonstrated that the species of Pythium that is contributing to the disease varies across orchards with a single dominant species commonly encountered at any specific orchard site^48^. In this study, *P. attrantheridium* (*Globisporangium attrantheridium*), *P. monospermum* (*Nematosporangium monospermum*) and *P. ultimum Globisporangium ultimum*) were the most relatively abundant oomycetal taxa, and *P. attrantheridium*, and *P. irregulare* (*Globisporangium irregulare*) are present at all ARD sites analyzed in this study. A previous study reported *Pythium irregulare* as one the main causal pathogens of ARD in the soil of five old orchards of the Annapolis Valley of Nova Scotia^19,20^.

Many bacterial families containing potential plant growth promotion taxa were part of the core microbiome. Some members of the family Chitinophagaceae, which demonstrated high relative abundance in orchard soils, have an ability to produce indole-3-acetic acid, solubilize phosphate, and possess ACC deaminase activity. Each of these attributes may help to promote plant growth^49^. The Sphingomonadaceae include genera with plant growth-promoting activities. Some of these genera produce phytohormones salicylic acid, gibberellins, indole-3-acetic acid, and abscisic acid^50^ and induce host-plant systemic resistance^51,52^. The presence of Solibacteraceae in the plant rhizosphere was linked to plant resistance to *Fusarium* pathogens^53^. Moreover, Actinobacteria and Pseudonocardiaceae were reported to exhibit antimicrobial ability against some bacteria and fungi^54^.

Microorganisms, which play significant role in the turnover of organic plant material, and soil fertility were also a part of the core microbiome. The *Xanthobacteraceae* family, contains potential nitrogen fixers, and degraders of alkenes, halogenated aliphatic and aromatic compounds, terpenes, thiophenes, or polyaromatic compounds^55^. Members the Nitrosomonadaceae family, contain species involved in nitrification, sulfur cycling, and plant growth promotion^56^. Many strains belonging to the Micromonosporaceae family can degrade chitin, cellulose, lignin, and pectin^57^. In addition to plant beneficial microorganisms, potential phytopathogens were found in the core microbiome.

Of the six genera of plant-parasitic nematodes found in the orchard soils, only root-lesion and dagger nematodes are known to be apple pests. Our analyses did not identify the species of root-lesion nematodes in each sample, but prior research has confirmed the widespread occurrence of *P. penetrans* in Nova Scotia orchards, including one of the orchards (KEN) sampled in this study^58^. Root-lesion nematodes are known to cause economically significant damage to apples on their own^59–61^and population densities of 30 to 100 *P. penetrans*/100 cm^3^ soil have been proposed as approximate damage thresholds for apple trees^59,62^. However, a recent field microplot study demonstrated apple growth reduction with an at-planting soil population density of 5.4 *P. penetrans*/100 cm^3^ soil^59^indicating that the *Pratylenchus* populations observed in these orchard soils would likely have measurable effects on apple growth. As migratory endoparasites of root cortical cells, *P. penetrans* cause cortical necrosis of fine feeder roots, making them vulnerable to infection by opportunistic fungal pathogens. Previous studies have demonstrated increased levels of infection or severity of disease caused by fungal pathogens including *Fusarium* sp., *Verticillium dahliae*, and *Rhizoctonia* sp. suggesting a synergistic relationship with fungal pathogens and increasing the severity of the broader replant disease complex^30,63^. Such interactions have not been demonstrated experimentally for apple, but it is worth noting that for strawberry, another perennial crop in the rosaceae, *P. penetrans* was demonstrated to increase infection of roots by *Rhizoctonia fragariae*, causing black root rot^64^.

The threshold for measurable dagger nematode damage to apples has been proposed to be 50 to 100 *Xiphinema*/100 cm^3^ soil (e.g. Nematode | Intermountain Fruit | USU; Nematodes - Ontario AppleIPM (gov.on.ca), and we speculate that they would have affected tree growth in the three soils with population densities of 40, 149 and 172 *Xiphinema*/100 cm^3^. *Xiphinema americanum* is also a vector of tomato ringspot virus which can be a problem in apple orchards. Pin nematodes are known to parasitize apple trees but only cause damage at much greater population densities than those found in these orchard soils (e.g. >500 *Paratylenchus*/100 cm^3^ soil). Ring nematodes, particularly the species *Mesocriconema xenoplax*, are known to be economically important parasites of *Prunus* fruit trees species^65^but they are not often reported from apple orchards at high population densities, and there are no recorded controlled-inoculation studies of their host-parasite relationship with apple. No species of spiral nematodes have been demonstrated to be apple pests and, similarly, no species of root-knot nematode known to exist in Canada that parasitize apples.

## Conclusions

Restrictions on the use of pre-plant, broad-spectrum soil fumigants, have resulted in the re-emergence of apple replant disease in Nova Scotia and across Canada. To effectively implement targeted and sustainable technologies and semi-selective agrochemicals for ARD management, a deeper understanding of disease etiology is essential. In this study, we investigated the soil microbial communities of six orchards with a long history of apple production by using next generation sequencing to gain insight into the nature of ARD complex. The results of this study show that ARD at these sites is primarily driven by biological factors rather than soil chemical or physical properties. We identified *Nectria ramulariae* (synonym *Cylindrocarpon ehrenbergii*) and *Ilyonectria robusta* (*Cylindrocarpon*-like asexual morphs) as putative causal pathogen of ARD, and as dominant fungal taxon in the soil microbiome at these sites. Among the Oomycota: we identified a high relative abundance of *P. attrantheridium* (*Globisporangium attrantheridium*), and *P. irregulare* (*Globisporangium irregulare*) at the ARD sites analyzed in this study. Additionally, the plant parasitic nematodes *Pratylenchus penetrans* and *Xiphinema americanum* were recovered at population levels consistent with potential economic damage to apple trees. A limitation of our study is that we did not find any direct correlation between microbial community variation and ARD severity. However, we would like to point out that one possible reason for this could be the limited range of disease severity across the sampled sites—five out of six sites exhibited severe ARD, while only one site showed a moderate level of severity. We did not have any sites with low ARD severity, which may have limited our ability to detect such correlations. A further limitation of this study is the absence of pathogen isolation and reinoculation experiments. Future research should focus on isolating, and identifying pathogens within the roots of young apple trees planted at these sites to confirm the potential pathogens identified in this study as causal agents. An additional complementary approach would be to apply functional metagenomics to identify the microbial activities and functional genes associated within the rhizosphere/roots or apple at ARD-affected sites. This could provide insights into the metabolic potential and ecological roles of soil microbes, including those involved in plant pathogenicity, nutrient cycling, and organic matter decomposition, thereby deepening our understanding of the functional dynamics underlying ARD. This approach will also enable resolution of the root microbiome at the species level and support the development of targeted disease management strategies.

## Materials and methods

### Site selection and collection of soils

Six orchard sites in the Annapolis Valley were chosen in fall 2020, representing the main locations of apple growing in the regions: (1) ROE (Rockland East area); (2) ROW (Rockland West area, ); (3) CAN (Canard area); (4) AYL (Aylesford area); (5) KEN (Kentville); (6) BER (Berwick area). In the Annapolis Valley of Nova Scotia, apples are produced under rain-fed (non-irrigated) conditions in a maritime climate with long cold winters, short summers and generally wet falls and springs. Droughty conditions can exist for short periods in the summer. In addition, soils are generally coarse, ranging from sandy to sandy loam in texture, with clay contents rarely exceeding 18% clay^66^ and, where they do, they are generally found in poorly drained depressions that collect run-off and seepage and are not suited to apple production. Soil classifications were based on the Agriculture Canada Expert Committee on Soil Survey^67^. At each site, five samples per site were collected from the 0–30 cm depth by sub-sampling 10 locations under the tree canopy on the herbicide strip along tree rows. The resulting samples (~ 2 L each) were combined, thoroughly blended, and passed through an 8 mm screen on-site to eliminate coarse organic material, stones and other debris from the sample. A composite soil sample was drawn from each sample for the following analyses: (i) a 300 mL sub-sample was stored at 4 °C and shipped to the Agriculture and Agri-Food Canada Summerland Research and Development Centre for nematode analysis; (ii) a 250 g sub-sample was stored at − 80 °C for DNA extraction; (iii) the remaining sample was dried, ground and sieved through a 2 mm screen, and analyzed for soil physio-chemical properties using standard methods. Information on soil fertility, orchard characteristics, and land and physical soil characteristics is provided in Tables 1 and 2. All tools and equipment were sterilized with 75% ethanol prior to sample collection at each site. The remaining bulk orchard samples were stored moist in totes lined with plastic at 5 °C and used for ARD bio-assays in greenhouse.

### Bioassays for ARD in the greenhouse

Apple seedlings were used in bio-assays to confirm the presence of ARD and establish the relative degree of severity between the experimental orchard soils. Apple seeds (Malus domestica Borkh. Var. Golden Delicious) were germinated in seedling trays using a pasteurized, soilless growing medium (Promix^®^), and grown for 3 weeks until the plants were ~ 8 cm high with a good root-ball. Seedlings were subsequently selected for uniformity and transplanted into 6” nursery pots (one per pot) containing pasteurized (p) or non-pasteurized (np) orchard soil, and grown in the greenhouse for 9 weeks (April 8–June 11, 2020). Pasteurization of the six orchard soils was accomplished by exposing the moist soil to a temperature of 70 °C for 2 h across 2 cycles, each 24 h apart. Greenhouse settings for the plant growth period were 22 °C and 18 °C for day and night temperatures, respectively. Day length during the spring growth period varied from 13 h on April 9 to 15.5 h on June 11. The trial was arranged in a randomized complete block design with 6 orchard soils, 2 levels of soil pasteurization (p, np) and 4 replications. All pots were watered frequently with de-chlorinated tap water and intermittently with complete, soluble nutrient solution at label rate. Aerial dry biomass accumulation after 8 weeks was used as the response variate to calculate seedling growth response to the pasteurization of soil as follows^32^:

$$\% {\text{ }}R{\text{ }} = {\text{ }}100_{*} \left( {x_{p} {-}{\text{ }}x_{{np}} } \right){\text{ }}/{\text{ }}x_{{np}} ,$$

where x_p_ and x_np_ are the aerial dry biomass accumulations for the p and np soil treatments respectively. We ranked the severity of ARD in the experimental orchards as Severe (% *R* > 100%), Moderate (% *R* = 50 to 100%), and Low (% *R* < 50%).

### DNA extraction and sequencing

DNA extraction from five samples per orchard from 250 mg of well homogenized soils was carried out using DNeasy PowerSoil Kit (cat #12888-100, Qiagen, Valencia, CA) according to the manufacturer’s protocol with slight modifications to increase the yield and obtain high-quality DNA^68^. In brief, the modification includes the use of buffer from the Power Bead tubes for washing the soil slurries into the Power Bead tubes. After adding 60 µl of solution C1 (step 2) the samples were incubated at 65 °C for 20 min to inactivate the DNases. Samples were then vortexed for 20 min (step 4) and incubated at 4 °C (step 7). In step 9 of the protocol, only 600 µl of supernatant was used, with 200 µl of C3 in step 10. DNA quality and concentration were measured using a NanoDrop 1000 spectrophotometer (Thermo Scientific, Waltham, USA). Isolate DNA was visualized on a 1% agarose gel (Invitrogen Ultrapure catalog number 16500-500) in 1X Tris-borate-EDTA buffer. To make sure the quality of DNA is good, PCR amplification was carried out with all the primer pairs that were used for subsequent meta amplicon sequencing. At least 50 ng (10µL) of DNA from each sample were sent to the Dalhousie University CGEB-IMR (http://cgeb-imr.ca/) for library preparation and sequencing. The bacterial V6-V8, 16 S rRNA gene was amplified using the primer set B969F (ACGCGHNRAACCTTACC) and BA1406R: (ACGGGCRGTGWGTRCAA)^69^and the fungal internal transcribed spacer ITS2 region was amplified using the primer pair ITS86(F): (GTGAATCATCGAATCTTTGAA): ITS4(R) (TCCTCCGCTTATTGATATGC)^70^. Samples were multiplexed using a dual-indexing approach and sequenced using an Illumina MiSeq with paired-end 2 × 300 bp reads. All PCR procedures and Illumina sequencing details were as previously described^71^. For oomycete sequencing, a sub sample of DNA was sent to Molecular Technologies Laboratory at AAFC Ottawa Research and Development Centre. For oomycetes, the ITS region (oomITS) was sequenced using primer Oom_SSU-ITS: CGGAAGGATCATTACCACAC and Oom_lo5.8S47C: ATTACGTATCGCAGTTCGCA^72^. Library preparation and sequencing was conducted as described above. All sequences generated in this study are available in the NCBI sequence read archive under the accession numbers PRJNA968027, PRJNA968028, and PRJNA968029.

### Sequencing data processing

Overlapping paired-end reads were stitched together using perl run_pear.pl script^73^. Paired sequences were then imported as a QIIME2 artifact^74^. The sequences were trimmed of their primers with Cutadapt^75^. Low-quality sequences were removed using QIIME2’s q-score-joined function. Sequences were organized into Amplicon Sequence Variants (ASV) with QIIME2’s Deblur plug-in^76^ using a trim length of 293 and 401 base. ASVs with a frequency < 0.1% of the total reads for the 16 S and ITS datasets were filtered out in order to compensate for MiSeq run-to-run bleed-through^71^. 16 S RNA ASVs were aligned with MAFFT v7.310^77^ to create de novo multiple sequence alignments, which were used to create a tree using FastTree v2.1.10^78^. 16 S rRNA and ITS ASVs were classified taxonomically using a Naïve-Bayes RDP classifier and accessing the SILVA rRNA^79^ and UNITE ITS database v7.2^80^, respectively. Reads annotated as mitochondria and chloroplast were filtered. For oomycetes reads processing, a standalone Nucleotide-Nucleotide BLAST+ (version 2.9.0+) search (mega blast) was performed to obtain the best high-scoring segment pair presenting at least 99% similarity and minimum 100-bp alignment length with the query sequence. Manually curated ITS sequences from Phytophthora*-*ID (http://phytophthora-id.org)^75,81^ were used as nucleotide reference database for BLAST. The annotation of all oomycetes ASVs was manually verified and updated using Nucleotide-Nucleotide BLAST against the NCBI nucleotide collection.

In brief, 166,769, 164,626 and 58,725 high-quality non-chimeric reads were obtained from 30 samples of 16 S rRNA, fungal ITS2 and oomycetes ITS (oomITS), respectively. These sequences were clustered into 5,174 (16 S rRNA), 1,026 (ITS2) and 282 (oomITS) ASVs. The datasets were rarefied to the depth of 1,215, 1,628 and 1,192 reads resulting in the datasets comprising 4,547 (16 S rRNA), 1,006 (ITS2) and 258 (oomITS) ASVs, respectively.

### Sequencing data analysis

Alpha-diversity (Chao1 richness, Simpson evenness and Shannon diversity) and beta-diversity metrics were generated using QIIME2. Variations in sample groupings explained by weighted unifrac beta-diversity distances (Adonis tests, 999 permutations) were run in QIIME2 to calculate how sample groupings are related to microbial community structure. Visualization was done using the ggplot2 package in R^82^. Differential relative abundance testing of classes and genera between orchard sites was performed using an analysis of composition of microbiomes (ANCOM) R package on non-rarefied ASV tables run in QIIME2 ^82^. Significant results were based on a q-value of 0.05. ASVs, which were found in ≥ 80% of all samples, were assigned as core ASVs using core function in QIIME2. Families present in ≥ 80% of samples across each orchard were assigned as core microbial families. Community-level patterns were analyzed using Redundancy Analysis (RDA) with the *vegan* R package in R (v2,6,-4)^83^.

### Nematode analysis

The wet sieving-sucrose centrifugation procedure^84^ was used to extract nematodes from triplicate 100 cm^3^ subsamples from each sample. The entire of extract was poured into a gridded counting dish and observed with a Meiji Techno TC5100 inverted microscope (Meiji Techno America, Campbell, CA, USA). Plant-parasitic nematodes in each extract were identified according to genus on the basis of morphological features observed at 400X magnification^85^and the number of nematodes of each genus was counted. Data were expressed as the number of nematodes of each genus per 100 cm^3^ soil, and averages of the triplicate extracts from each sample are reported in Table 5.

27.The multiple personalities of Streptomyces spp. from the rhizosphere of apple cultivated in brassica seed meal ameded soils.

## Supplementary Information

Below is the link to the electronic supplementary material.

Supplementary Material 1

Supplementary Material 2

Supplementary Material 3

## Data Availability

The datasets generated in the current study are available in the [SRA NCBI] repository, and can be accessed from the following link (https://www.ncbi.nlm.nih.gov/ sra/PRJNA968027, PRJNA968028 and PRJNA968029).

## Declarations

### Statement regarding research involving plants

All experiments were compiled with AAFC and international guidelines on research on plants.

## References

[1] Mai, W. F. & GS, A. Controlling replant diseases of pome and stone fruits in Northeastern united States by Preplant fumigation. *Plant Dis.* **65**, 859–864 (1981).

[2] Braun, P. G., Fuller, K. D., McRae, K. & Fillmore, S. A. Response of ‘Honeycrisp^®^’apple trees to combinations of pre-plant fumigation, deep ripping, and hog manure compost incorporation in a soil with replant disease. *HortScience* **45**, 1702–1707 (2010).

[3] Tewoldemedhin, Y. T., Mazzola, M., Botha, W. J., Spies, C. F. & McLeod, A. Characterization of fungi (*Fusarium* and *Rhizoctonia*) and oomycetes (*Phytophthora* and *Pythium*) associated with Apple orchards in South Africa. *Eur. J. Plant Pathol.* **130**, 215–229 (2011).

[4] DuPont, S. T., Hewavitharana, S. S. & Mazzola, M. Field scale application of brassica seed meal and anaerobic soil disinfestation for the control of Apple replant disease. *Appl. Soil. Ecol.* **166** (2021).

[5] Auvil, T. et al. In *IX International Symposium on Integrating Canopy, Rootstock and Environmental Physiology in Orchard Systems* 903, 265–271 (2008).

[6] Tewoldemedhin, Y. T., Mazzola, M., Labuschagne, I. & McLeod, A. Biochemistry A multi-phasic approach reveals that Apple replant disease is caused by multiple biological agents, with some agents acting synergistically. *Soil. Biol*. **43**, 1917–1927 (2011).

[7] Yin, C. et al. How to plant Apple trees to reduce replant disease in Apple orchard: a study on the phenolic acid of the replanted Apple orchard. *PloS One*. **11**, e0167347 (2016). 27907081 10.1371/journal.pone.0167347 PMC5132267

[8] Yin, C. et al. The research advance on Apple replant disease. *Acta Horticulturae Sinica*. **44**, 2215 (2017).

[9] Browne, G., Connell, J. & Schneider, S. Almond replant disease and its management with alternative pre-plant soil fumigation treatments and rootstocks. *Plant Dis.* **90**, 869–876 (2006). 30781023 10.1094/PD-90-0869

[10] Manici, L. et al. Relationship between root-endophytic microbial communities and replant disease in specialized Apple growing areas in Europe. *Appl. Soil. Ecol.* **72**, 207–214 (2013).

[11] Spath, M. et al. Linking soil biotic and abiotic factors to Apple replant disease: a greenhouse approach. *J. Phytopathol.* **163**, 287–299 (2015).

[12] Yim, B., Smalla, K. & Winkelmann, T. Evaluation of Apple replant problems based on different soil disinfection treatments—links to soil microbial community structure? *Plant. Soil.* **366**, 617–631 (2013).

[13] Mazzola, M. Elucidation of the microbial complex having a causal role in the development of Apple replant disease in Washington. *Phytopathology* **88**, 930–938 (1998). 18944871 10.1094/PHYTO.1998.88.9.930

[14] Balbín-Suárez, A. et al. Exploring microbial determinants of Apple replant disease (ARD): A microhabitat approach under split-root design. *FEMS Microbiol. Ecol.* **96** (2020). 10.1093/femsec/fiaa211 33045057

[15] Tilston, E. et al. Candidate causal organisms for Apple replant disease in the UK. *Phytobiomes J.* **2** (2018).

[16] Lucas, M., Balbín-Suárez, A., Smalla, K. & Vetterlein, D. Root growth, function and rhizosphere Microbiome analyses show local rather than systemic effects in Apple plant response to replant disease soil. *PLoS One*. **13**, e0204922 (2018). 30296282 10.1371/journal.pone.0204922 PMC6175279

[17] Van Schoor, L., Denman, S. & Cook, N. Characterisation of Apple replant disease under South African conditions and potential biological management strategies. *Sci. Hort.* **119**, 153–162 (2009).

[18] Manici, L., Ciavatta, C., Kelderer, M. & Erschbaumer, G. Soil replant problems in South tyrol: role of fungal pathogens and microbial population in conventional and organic Apple orchards. *Plant. Soil.* **256**, 315–324 (2003).

[19] Braun, P. The combination of *Cylindrocarpon lucidum* and *Pythium irregulare* as a possible cause of Apple replant disease in Nova Scotia. *Can. J. Plant Pathol.* **13**, 291–297 (1991).

[20] Braun, P. Effects of *Cylindrocarpo*n and *Pythium* species on Apple seedlings and potential role in Apple replant disease. *Can. J. Plant Pathol.* **17**, 336–341 (1995).

[21] Manici, L. et al. Relationship between root-endophytic microbial communities and replant disease in specialized Apple growing areas in Europe. *Appl. Soil. Ecol.* **72**, 207–214 (2013).

[22] Kelderer, M., Manici, L. M., Caputo, F. & Thalheimer, M. Soil planting in the ‘inter-row’to overcome replant disease in Apple orchards: a study on the effectiveness of the practice based on microbial indicators. *Plant. Soil.* **357**, 381–393 (2012).

[23] Mazzola, M. Identification and pathogenicity of *Rhizoctonia* spp. Isolated from Apple roots and orchard soils. *Phytopathology* **87**, 582–587 (1997). 18945073 10.1094/PHYTO.1997.87.6.582

[24] Franke-Whittle, I. H., Manici, L. M., Insam, H. & Stres, B. Soil rhizosphere bacteria and fungi associated with plant growth in soils of three replanted Apple orchards. *Plant. Soil.* **395**, 317–333 (2015).

[25] Winkelmann, T. et al. Apple replant disease: causes and mitigation strategies. *Curr. Issues Mol. Biol.* **30**, 89–106 (2019). 30070653 10.21775/cimb.030.089

[26] Gu, Y. H. & Mazzola, M. Modification of fluorescent pseudomonad community and control of Apple replant disease induced in a wheat cultivar-specific manner. *Appl. Soil. Ecol.* **24**, 57–72 (2003).

[27] Mazzola, M., Zhao, X., Tewoldemedhin, Y. & Mcleod, A. The multiple personalities of streptomyces spp. From the rhizosphere of Apple cultivated in brassica seed meal ameded soils in phytopathology, **99** S150 (2009).

[28] Manici, L. & Caputo, F. Fungal community diversity and soil health in intensive potato cropping systems of the East Po valley, Northern Italy. *Ann. Appl. Biol.* **155**, 245–258 (2009).

[29] Xu, L. et al. Soil fungal community structure along a soil health gradient in pea fields examined using deep amplicon sequencing. *Soil. Biol*. **46**, 26–32 (2012).

[30] Mazzola, M. & Manici, L. M. Apple replant disease: role of microbial ecology in cause and control. *Annu. Rev. Phytopathol.* **50**, 45–65 (2012). 22559069 10.1146/annurev-phyto-081211-173005

[31] Liu, J., Zhang, W., Li, Y., Sun, Y. & Bian, X. Effects of long-term continuous cropping system of cotton on soil physical-chemical properties and activities of soil enzyme in Oasis in Xinjiang. *Scientia Agricultura Sinica*. **42**, 725–733 (2009).

[32] Gongshuai, W. et al. Analysis of the fungal community in Apple replanted soil around Bohai Gulf. *Hortic. Plant. J.* **4**, 175–181 (2018).

[33] Li, J. J. et al. Effects of *Malus hupehensis* seedlings and *Allium fistulosum* mixed cropping on replanted soil environment. *Acta Horticulturae Sinica*. **43**, 1853–1862 (2016).

[34] Barrios, E. Soil biota, ecosystem services and land productivity. *Ecol. Econ.* **64**, 269–285 (2007).

[35] Liu, X. et al. Microbial community diversities and taxa abundances in soils along a seven-year gradient of potato monoculture using high throughput pyrosequencing approach. *PloS One*. **9**, e86610 (2014). 24497959 10.1371/journal.pone.0086610 PMC3907449

[36] Singh, B. K. et al. Plant pathogens, microbiomes, and soil health. *Trends Microbiol.* (2025). 10.1016/j.tim.2025.03.013 40274492

[37] Westcott, I. I. I., Beer, S., Israel, H. J. P. & S. & Interactions between actinomycete-like organisms and young Apple roots grown in soil conductive to Apple replant disease. *Phtopathology* **77**, 1071–1077 (1987).

[38] Xue, C. et al. Manipulating the banana rhizosphere Microbiome for biological control of Panama disease. *Sci. Rep.* **5**, 1–11 (2015). 10.1038/srep11124 PMC4525139 26242751

[39] Soman, A. G., Gloer, J. B. & Wicklow, D. T. Antifungal and antibacterial metabolites from a Sclerotium-Colonizing isolate of Mortierella v inacea. *J. Nat. Prod.* **62**, 386–388 (1999). 10075797 10.1021/np980411h

[40] Shalapy, N. M. & Kang, W. *Fusarium oxysporum* & *Fusarium solan*i: Identification, Characterization, and Differentiation the Fungal Phenolic Profiles by HPLC and the Fungal Lipid Profiles by GC-MS. *J. Food Qual.* **2022**, 4141480 (2022).

[41] Bacon, C. W. & Yates, I. E. Endophytic root colonization by *Fusarium* species: histology, plant interactions, and toxicity. *Microb. Root Endophytes*. 133–152 (2006).

[42] Macia-Vicente, J. G. et al. Fungal root endophytes from natural vegetation in mediterranean environments with special reference to *Fusarium* spp. *FEMS Microbiol. Ecol.* **64**, 90–105 (2008). 18248439 10.1111/j.1574-6941.2007.00443.x

[43] Manici, L. M. & Caputo, F. Soil fungal communities as indicators for replanting new Peach orchards in intensively cultivated areas. *Eur. J. Agron.* **33**, 188–196 (2010).

[44] Shin, S. et al. Transcriptome changes specifically associated with Apple (Malus domestica) root defense response during *Pythium ultimum* infection. *Physiol. Mol. Plant. Pathol.* **94**, 16–26 (2016).

[45] Dullahide, S., Stirling, G., Nikulin, A. & Stirling, A. The role of nematodes, fungi, bacteria, and abiotic factors in the etiology of Apple replant problems in the granite belt of Queensland. *Aust. J. Exp. Agric.* **34**, 1177–1182 (1994).

[46] Chaverri, P., Salgado, C., Hirooka, Y., Rossman, A. & Samuels, G. Delimitation of neonectria and cylindrocarpon (Nectriaceae, hypocreales, Ascomycota) and related genera with cylindrocarpon-like anamorphs. *Stud. Mycol.* **68**, 57–78 (2011). 21523189 10.3114/sim.2011.68.03 PMC3065985

[47] Tewoldemedhin, Y. T., Mazzola, M., Mostert, L. & McLeod, A. *Cylindrocarpon* species associated with Apple tree roots in South Africa and their quantification using real-time PCR. *Eur. J. Plant Pathol.* **129**, 637–651 (2011).

[48] Mazzola, M., Andrews, P. K., Reganold, J. P. & Levesque, C. A. Frequency, virulence, and Metalaxyl sensitivity of *Pythium* spp. Isolated from Apple roots under conventional and organic production systems. *Plant Dis.* **86**, 669–675 (2002). 30823243 10.1094/PDIS.2002.86.6.669

[49] Madhaiyan, M. et al. Arachidicoccus rhizosphaerae gen. Nov., sp. Nov., a plant-growth-promoting bacterium in the family chitinophagaceae isolated from rhizosphere soil. *Int. J. Syst. Evol. MicroBiol.* **65**, 578–586 (2015). 25404481 10.1099/ijs.0.069377-0

[50] Yang, S. et al. Growth-promoting sphingomonas paucimobilis ZJSH1 associated with dendrobium officinale through phytohormone production and nitrogen fixation. *Microb. Biotechnol.* **7**, 611–620 (2014). 25142808 10.1111/1751-7915.12148 PMC4265079

[51] Chapelle, E., Mendes, R., Bakker, P. A. & Raaijmakers, J. M. Fungal invasion of the rhizosphere Microbiome. *ISME J.* **10**, 265–268 (2016). 26023875 10.1038/ismej.2015.82 PMC4681858

[52] Hahm, M. S. et al. Biological control and plant growth promoting capacity of rhizobacteria on pepper under greenhouse and field conditions. *J. Microbiol.* **50**, 380–385 (2012). 22752900 10.1007/s12275-012-1477-y

[53] Mendes, L. W., Raaijmakers, J. M., de Hollander, M., Mendes, R. & Tsai, S. M. Influence of resistance breeding in common bean on rhizosphere Microbiome composition and function. *ISME J.* **12**, 212–224 (2018). 29028000 10.1038/ismej.2017.158 PMC5739014

[54] Chaouch, F. C. & Planomonospora Saccharothrix and actinophytocola genera in saharan soils of algeria: isolation, taxonomic identification and antagonistic properties. *J. Microbiol. Biotechnol. Food Sci.* 505–510 (2018).

[55] Oren, A. The family Xanthobacteraceae. In *The Prokaryotes* (eds Rosenberg, E., DeLong, E. F., Lory, S., Stackebrandt, E. & Thompson, F.) 709–726 (Springer, 2014).

[56] Prosser, J. I., Head, I. M. & Stein, L. Y. The family Nitrosomonadaceae. In *The Prokaryotes: Alphaproteobacteria and Betaproteobacteria*. (eds. E. Rosenberg, E.F. DeLong, S. Lory, E. Stackebrandt & F. Thompson) 901–918 (Springer, 2014).

[57] Trujillo, M. E., Hong, K. & Genilloud, O. The family Micromonosporaceae. In *The Prokaryotes* (eds Rosenberg, E., DeLong, E. F., Lory, S., Stackebrandt, E. & Thompson, F.) 499–569 (Springer, 2014).

[58] Forge, T., Neilsen, D., Neilsen, G. & Blatt, S. Dynamics of the impacts of *Pratylenchus penetrans* on Gisela^®^ Cherry rootstocks. *J. Nematol*. **51**, 1–10 (2019). 31088020 10.21307/jofnem-2019-008 PMC6929660

[59] King, L., Munro, P., Xu, H., Jones, M. & Forge, T. The Root-Lesion Nematode, Pratylenchus penetrans, Affects Early Growth and Physiology of Grafted M.9, G.41, and G.935 Apple Rootstocks Similarly Under Field Microplot Conditions. *Plant Dis.* **108** (2024). 10.1094/PDIS-10-23-2027-RE 38213117

[60] Bélair, G., Dauphinais, N. & Fournier, Y. Pathogenicity of *Pratylenchus penetran*s to dwarfing Apple rootstocks. *Phytoprotection*. **99**, 12–14 (2019).

[61] Ark, P. & Thomas, H. *Anguillulina pratensis* in relation to root injury of Apple and other fruit trees. *Phytopathology* **26**, 1128–1134 (1936).

[62] Mazzola, M., Brown, J., Zhao, X., Izzo, A. D. & Fazio, G. Interaction of brassicaceous seed meal And Apple rootstock on recovery of pythium spp. And Pratylenchus penetrans from roots grown in replant soils. *Plant Dis.* **93**, 51–57 (2009). 30764268 10.1094/PDIS-93-1-0051

[63] Castillo, P. & Vovlas, N. *Pratylenchus (Nematoda: Pratylenchidae): Diagnosis, Biology, Pathogenicity and Management*, vol. 6 (Brill, 2007).

[64] LaMondia, J. Interaction of Pratylenchus penetrans and rhizoctonia fragariae in strawberry black root rot. *J. Nematol*. **35**, 17 (2003). 19265969 PMC2620604

[65] Ferris, H., McKenry, M., Jaffee, B., Anderson, C. & Juurma, A. Population characteristics and dosage trajectory analysis for *Mesocriconema Xenoplax* in California Prunus orchards. *J. Nematol*. **36**, 505 (2004). 19262832 PMC2620792

[66] Holmstrom, D. A. & Thompson, B. *Soils of the Annapolis Valley Area of Nova Scotia*, vol. 89 (Agriculture Development Branch, 1989).

[67] Group, C., Agriculture, C. & Branch, A. F. C. R. *The Canadian System of Soil Classification* (NRC Research, 1998).

[68] Wright, A. H. et al. A characterization of a cool-climate organic vineyard’s Microbiome. *Phytobiomes J.* **6**, 69–82 (2022).

[69] Comeau, A. M., Li, W. K., Tremblay, J. É., Carmack, E. C. & Lovejoy, C. Arctic ocean microbial community structure before and after the 2007 record sea ice minimum. *PLoS One*. **6**, e27492 (2011). 22096583 10.1371/journal.pone.0027492 PMC3212577

[70] De Op, M. et al. Comparison and validation of some ITS primer pairs useful for fungal metabarcoding studies. *PLoS One*. **9**, e97629 (2014). 24933453 10.1371/journal.pone.0097629 PMC4059633

[71] Comeau, A. M., Vincent, W. F., Bernier, L. & Lovejoy, C. Novel Chytrid lineages dominate fungal sequences in diverse marine and freshwater habitats. *Sci. Rep.* **6**, 30120 (2016). 27444055 10.1038/srep30120 PMC4957111

[72] Man, Veld, W. A., de Cock, A. W. & Ilieva, E. André lévesque gene flow analysis of *Phytophthora Porri* reveals a new species: *Phytophthora brassicae* sp. nov. *Eur. J. Plant Pathol.* **108**, 51–62 (2002).

[73] Zhang, J., Kobert, K., Flouri, T. & Stamatakis, A. PEAR: a fast and accurate illumina Paired-End read merger. *Bioinformatics* **30**, 614–620 (2014). 24142950 10.1093/bioinformatics/btt593 PMC3933873

[74] Bolyen, E. et al. QIIME 2: reproducible, interactive, scalable, and extensible Microbiome data science. *PeerJ Preprints*. **6**, e27295 (2018). 10.1038/s41587-019-0209-9 PMC7015180 31341288

[75] Grünwald, N. J. et al. *Phytophthora*-ID. Org: a sequence-based *Phytophthora* identification tool. *Plant Dis.* **95**, 337–342 (2011). 30743500 10.1094/PDIS-08-10-0609

[76] Comeau, A. M., Douglas, G. M. & Langille, M. G. Microbiome helper: a custom and streamlined workflow for microbiome research. *mSystems*. **2** (2017). 10.1128/mSystems.00127-16 PMC5209531 28066818

[77] Katoh, K. & Standley, D. M. MAFFT multiple sequence alignment software version 7: improvements in performance and usability. *Mol. Biol. Evol.* **30**, 772–780 (2013). 23329690 10.1093/molbev/mst010 PMC3603318

[78] Price, M. N., Dehal, P. S. & Arkin, A. P. FastTree 2-approximately maximum-likelihood trees for large alignments. *PLoS One*. **5**, e9490 (2010). 20224823 10.1371/journal.pone.0009490 PMC2835736

[79] Quast, C. et al. The SILVA ribosomal RNA gene database project: improved data processing and web-based tools. *Nucleic Acids Res.* **41**, D590–D596 (2012). 23193283 10.1093/nar/gks1219 PMC3531112

[80] Nilsson, R. H. et al. The UNITE database for molecular identification of fungi: handling dark taxa and parallel taxonomic classifications. *Nucleic Acids Res.* **47**, D259–D264 (2019). 30371820 10.1093/nar/gky1022 PMC6324048

[81] Robideau, G. P. et al. DNA barcoding of oomycetes with cytochrome c oxidase subunit I and internal transcribed spacer. *Mol. Ecol. Resour.* **11**, 1002–1011 (2011). 21689384 10.1111/j.1755-0998.2011.03041.x PMC3195333

[82] Gómez-Rubio, V. ggplot2-elegant graphics for data analysis. *J. Stat. Softw.* **77**, 1–3 (2017).

[83] Oksanen, J. & _vegan Community Ecology Package_. R package version 2.6–4. *Community Ecology Package. R package version 2.6-4* (2022). https://CRAN.R-project.org/package=vegan.

[84] Carter, M. R. & Gregorich, E. G. *Soil Sampling and Methods of Analysis* (CRC, 2007).

[85] Mai, W. *Plant-parasitic Nematodes: a Pictorial Key To Genera* (Cornell University Press, 2018).

