# Supplementary material for: Exploring soil microbial and plant parasitic nematode communities involved in the apple replant disease complex in Nova Scotia
Source: Sci Rep. 2025 Oct 2;15:34402. doi: 10.1038/s41598-025-17349-8 (PMC12491575; doi:10.1038/s41598-025-17349-8)
Supplement: Supplementary file 1 — Supplementary Material 1 [file 41598_2025_17349_MOESM1_ESM.pptx]

## Slide 1
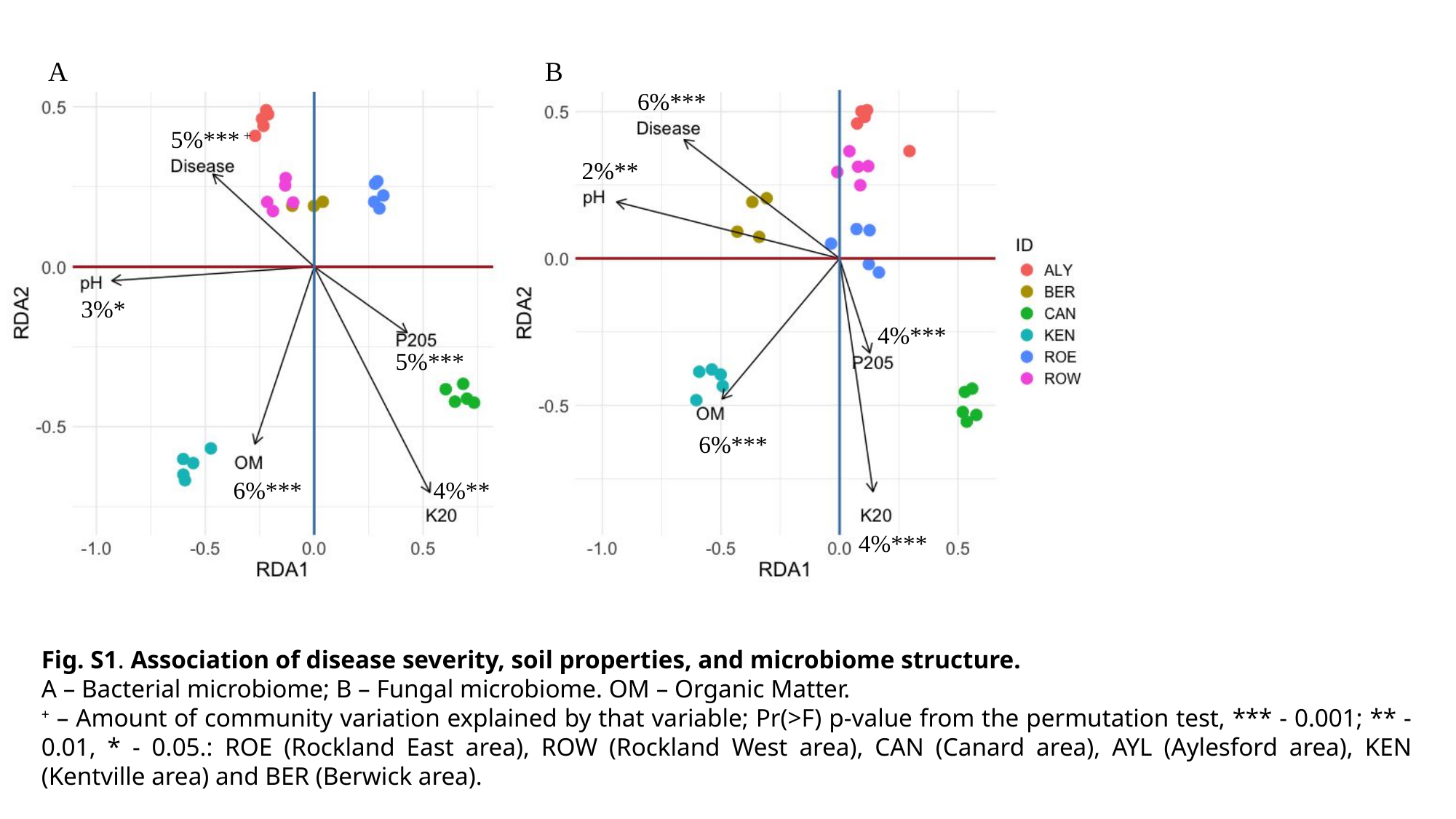

A
B
6%***
5%*** +
2%**
3%*
4%***
5%***
6%***
4%**
6%***
4%***
Fig. S1. Association of disease severity, soil properties, and microbiome structure.
A – Bacterial microbiome; B – Fungal microbiome. OM – Organic Matter.
+ – Amount of community variation explained by that variable; Pr(>F) p-value from the permutation test, *** - 0.001; ** - 0.01, * - 0.05.: ROE (Rockland East area), ROW (Rockland West area), CAN (Canard area), AYL (Aylesford area), KEN (Kentville area) and BER (Berwick area).

## Slide 2
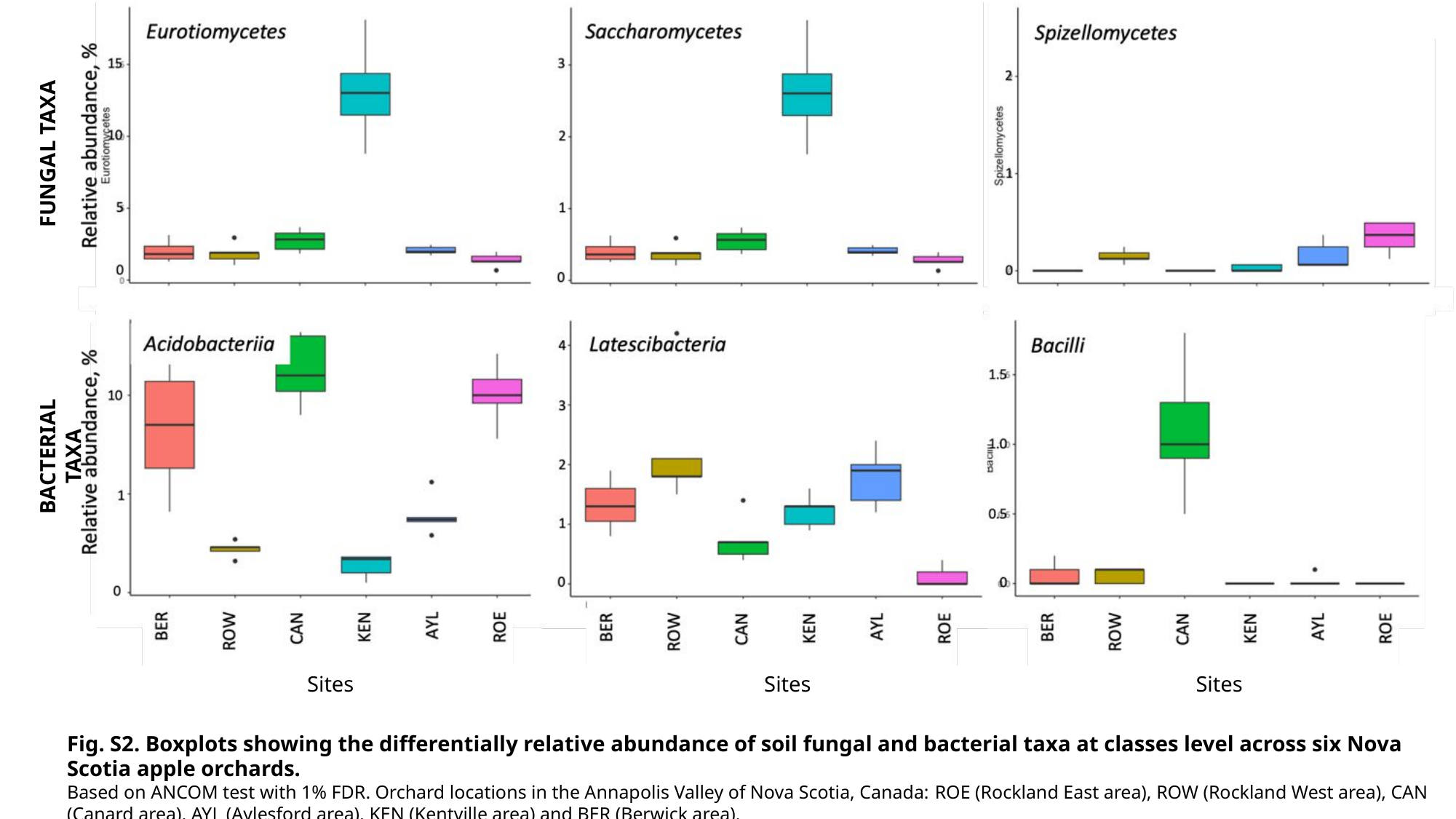

FUNGAL TAXA
BACTERIAL TAXA
Sites
Sites
Sites
Fig. S2. Boxplots showing the differentially relative abundance of soil fungal and bacterial taxa at classes level across six Nova Scotia apple orchards.
Based on ANCOM test with 1% FDR. Orchard locations in the Annapolis Valley of Nova Scotia, Canada: ROE (Rockland East area), ROW (Rockland West area), CAN (Canard area), AYL (Aylesford area), KEN (Kentville area) and BER (Berwick area).
