# Supplementary material for: Exploring soil microbial and plant parasitic nematode communities involved in the apple replant disease complex in Nova Scotia
Source: Sci Rep. 2025 Oct 2;15:34402. doi: 10.1038/s41598-025-17349-8 (PMC12491575; doi:10.1038/s41598-025-17349-8)
Supplement: Supplementary file 2 — Supplementary Material 2 [file 41598_2025_17349_MOESM2_ESM.docx]

**run_pear.pl:**

run_pear.pl script was run by running

perl run_pear.pl -p 40 -o stitched_reads raw_data/*

On UNIX environment

run_pear.pl was installed from

https://cme.h-its.org/exelixis/web/software/pear/

**The script to visualize differentially represented fungal taxa using ggplot boxplot in input file** Box_plot_ITS_IN.txt**:**

library(ggplot2)

setwd("/boxplot")

test <- read.table( file="Box_plot_ITS_IN.txt", header=T, sep="\t", row.names=1 )

test$Block_1 <- as.factor(test$Block_1)

class(test$Block_1)

p <- ggplot(test, aes(x=Block_1, y=Lasiosphaeris, fill=Block_1)) +

theme(axis.text.x=element_blank(), axis.title.x=element_blank()) +

geom_boxplot()

p + theme_classic() + theme(legend.position = "none")

p <- ggplot(test, aes(x=Block_1, y=Chaetothyriales, fill=Block_1)) +

theme(axis.text.x=element_blank(), axis.title.x=element_blank()) +

geom_boxplot()

p + theme_classic() + theme(legend.position = "none")

p <- ggplot(test, aes(x=Block_1, y=Peziza, fill=Block_1)) +

theme(axis.text.x=element_blank(), axis.title.x=element_blank()) +

geom_boxplot()

p + theme_classic() + theme(legend.position = "none")

p <- ggplot(test, aes(x=Block_1, y=Mariannaea, fill=Block_1)) +

theme(axis.text.x=element_blank(), axis.title.x=element_blank()) +

geom_boxplot()

p + theme_classic() + theme(legend.position = "none")

p <- ggplot(test, aes(x=Block_1, y=Matsushimamyces, fill=Block_1)) +

theme(axis.text.x=element_blank(), axis.title.x=element_blank()) +

geom_boxplot()

p + theme_classic() + theme(legend.position = "none")

p <- ggplot(test, aes(x=Block_1, y=Chaetothyriales_unidentified, fill=Block_1)) +

theme(axis.text.x=element_blank(), axis.title.x=element_blank()) +

geom_boxplot()

p + theme_classic() + theme(legend.position = "none")

p <- ggplot(test, aes(x=Block_1, y=Cyberlindnera, fill=Block_1)) +

theme(axis.text.x=element_blank(), axis.title.x=element_blank()) +

geom_boxplot()

p + theme_classic() + theme(legend.position = "none")

p <- ggplot(test, aes(x=Block_1, y=Chaetomiaceae, fill=Block_1)) +

theme(axis.text.x=element_blank(), axis.title.x=element_blank()) +

geom_boxplot()

p + theme_classic() + theme(legend.position = "none")

p <- ggplot(test, aes(x=Block_1, y=Cordycipitaceae, fill=Block_1)) +

theme(axis.text.x=element_blank(), axis.title.x=element_blank()) +

geom_boxplot()

p + theme_classic() + theme(legend.position = "none")

p <- ggplot(test, aes(x=Block_1, y=Paraconiothyrium, fill=Block_1)) +

theme(axis.text.x=element_blank(), axis.title.x=element_blank()) +

geom_boxplot()

p + theme_classic() + theme(legend.position = "none")

p <- ggplot(test, aes(x=Block_1, y=Onygenales, fill=Block_1)) +

theme(axis.text.x=element_blank(), axis.title.x=element_blank()) +

geom_boxplot()

p + theme_classic() + theme(legend.position = "none")

p <- ggplot(test, aes(x=Block_1, y=Neobulgaria, fill=Block_1)) +

theme(axis.text.x=element_blank(), axis.title.x=element_blank()) +

geom_boxplot()

p + theme_classic() + theme(legend.position = "none")
